# Draft Genome Sequence of *Prevotella copri* Isolated from the Gut of a Healthy Indian Adult

**DOI:** 10.1128/genomeA.00834-17

**Published:** 2017-09-14

**Authors:** Satyabrata Bag, Tarini Shankar Ghosh, Bhabatosh Das

**Affiliations:** Molecular Genetics Laboratory, Centre for Human Microbial Ecology, Translational Health Science and Technology Institute, NCR Biotech Science Cluster, Faridabad, India

## Abstract

*Prevotella copri*, a Gram-negative anaerobic rod-shaped bacterium, is frequently associated with the human gastrointestinal tract and influences host physiology, immunity, and metabolic pathways. In the present study, we report the draft genome sequence of *P. copri* isolated from the gut of a healthy Indian adult.

## GENOME ANNOUNCEMENT

*Prevotella copri* is the most dominant bacterial species in the microbial community inhabiting the gastrointestinal tracts of the Indian population (1–3). The genome of *P. copri* is enriched with hundreds of glycoside hydrolase- and polysaccharide lyase-encoding genes that are not encoded by the host genome (4). However, the expansion of intestinal *P. copri* may also enhance susceptibility to disorders like arthritis (5). To understand the physiology and metabolic potential of *P. copri*, it is important to explore its functional repertoire by decoding its genome sequence.

*P. copri* strain Indica was isolated from stool samples from a healthy adult Indian subject. Fresh stool sample was immediately resuspended in prereduced phosphate-buffered saline (PBS), serially diluted, and then plated onto a Wilkins Chalgren Anaerobic Hi Veg (WCAHV) agar base plate supplemented with nonspore anaerobic supplement and 5% (vol/vol) sterile defibrinated sheep blood. The plates were incubated for 24 h at 37°C in an anaerobic workstation (Whitley DG250) filled with 80% N_2_–10% CO_2_–10% H_2_ gases.

Genomic DNA was isolated from a colony of *P. copri* grown in WCAHV broth for 12 h using the THSTI method (6). Approximately 1 µg of genomic DNA was nebulized at 15 lb/in^2^ for 1 min for fragmentation of genomic DNA (1,400 to 1,800 bp). The nebulized DNA was purified by a PCR purification kit (Qiagen, USA) and end polished with T4 DNA polymerase, polynucleotide kinase, and *Taq* DNA polymerase (Roche, USA). A sequencing adaptor was ligated to the polished DNA fragments using DNA ligase (Roche). The genomic DNA libraries thus obtained were then purified using AMPure XP beads (Beckman Coulter, Inc., USA). The quality of the DNA libraries was analyzed using a high-sensitivity DNA chip in the 2100 Bioanalyzer (Agilent, USA). The total amount of double-stranded DNA (dsDNA) in the libraries was quantified using Picogreen dye in a Qubit fluorometer (Invitrogen, USA). About 140 × 10^7^ DNA library molecules per sample were clonally amplified by emulsion PCR in Mastercycler proS PCR systems (Eppendorf, Germany), purified using REMe integration (Roche) on Biomek 3000 instrument (Beckman Coulter, Inc.), and pyrosequenced in picotiter plates in GS-FLX+ genome sequencers (Roche).

*De novo* assembly of 498,385 reads, with modal read length of 901 bp and 45.4% GC content, was performed using the NCBI Prokaryotic Genome Annotation Pipeline (2013 release). The analysis of 3.92 Mbp sequences identified 3,303 genes, including 3,209 coding sequences (CDS) and 94 RNA-encoding genes. The genome sequence of strain Indica will contribute to a better understanding of the biology of *P. copri* and the molecular basis of its dominance in the gut of Indian subjects.

### Accession number(s).

The whole-genome shotgun project has been deposited at DDBJ/ENA/GenBank under the accession number NMPZ00000000. The version described in this paper is version NMPZ01000000. The whole-genome shotgun scaffolds are also available in the Sequence Read Archive database (SRS2338962).
